# PMTCT Data Management and Reporting during the Transition Phase of Implementing the Rationalised Registers in Amathole District, Eastern Cape Province, South Africa

**DOI:** 10.3390/ijerph192315855

**Published:** 2022-11-28

**Authors:** Oyebanji G. Oyebola, Jackson Debra, Mathole Thubelihle

**Affiliations:** 1 School of Public Health, University of the Western Cape, Cape Town 7535, South Africa; 2 London School of Hygiene and Tropical Medicine, London WC1E 7HT, UK

**Keywords:** South Africa, PMTCT, rationalisation of register, district health information system, documentation

## Abstract

Background: The National Department of Health, in March 2015, launched the implementation of Rationalisation of Register, aimed at reducing the amount of time invested in completing the registers and collecting data. Therefore, the number of registers used in the South African healthcare facilities was reduced from 56 to 6. Objectives: This study explored the effect of the rollout of Rationalisation of Register on the documentation and reporting of Prevention of Mother-to-Child Transmission (PMTCT) programme data with the existing source documents during the transitional period, especially with routine data collected and reported at various health care system levels. Methods: A mixed-method research approach was used, and three source documents, namely: Tally sheet, Antenatal care (ANC) register, and Tick register used for collecting and reporting PMTCT data, were reviewed. An in-depth interview was conducted with healthcare workers in four sub-districts of the Amathole district, Eastern Cape province of South Africa. Results: All selected facilities completed the three source documents. The facilities consolidated their PMTCT data monthly before reporting to the District Health Information System (DHIS). Less than half of the facilities had already started using the rationalised registers. However, they did not transition entirely because they still use other registers, especially the ANC register. Reasons for not displaying facility performance include clinicians not properly completing the clients’ information, and a shortage of staff to collect, report, and analyse data. Conclusions: PMTCT data management and reporting were challenging during the transitioning phase of implementing the rationalised registers because of different timelines instituted in the facilities and non-availability of source documents in some facilities. Capacity of the clinic staff involved in data collection should be built on programme care pathways, data monitoring, data capturing into the Routine Health Information System and complemented with coaching, mentoring, and supportive supervision for improved programme outputs and outcomes.

## 1. Introduction

The South African National Department of Health (NDoH), in March 2015, launched the implementation of Rationalisation of Register (ROR), which was aimed at reducing the amount of time invested in completing the registers and collecting data so that the health care workers would dedicate more time to improve data quality [1]. This forms part of the Continuous Quality Improvement (CQI) process implemented in the facilities which allows collection and reporting of quality data that is analyzed and used to continuously improve programme performance and delivery of quality services.

The decision that informed the implementation of ROR was based on the summary report of the national health care facility baseline audit conducted by Health Systems Trust (one of the department of health supporting partners) in 2012 where a couple of factors that affects the quality and use of data were identified [1]. One of such is poor quality of data collected within facilities and the belief that much of the information lacks credibility. The poor quality of data was evident in widely differing findings for certain data elements between geographical areas and over time. This arises from poorly designed data collection processes at facility level where there are many different forms and registers scattered across the facility, from which over-stretched or demotivated nurses have to extract data [1]. Another challenge at the facility level is that nurses may forget to capture information on every patient that presents for care as a result of the multiple roles they play. These may lead to over reporting; hence it is difficult to keep accurate statistics, and this does not give a true reflection of care given in the facility. Thereafter, recommendations were made that the number of registers used in the facilities be reduced from 56 to 6. The six standardised registers are Primary Health Care (PHC) Daily Tick Register plus Headcount Register, Tuberculosis (TB) Registers (for ETR.Net), antiretroviral therapy (ART) Register (for TIER.Net), Delivery Register, Theatre Register (including Medical Male Circumcision [MMC] and termination of pregnancy [TOP]) and Midnight Census Register.

Published papers evaluating selected aspects of the District Health Information System (DHIS) [2,3,4,5] found significant discrepancies in the completeness and accuracy of data, largely due to poor documentation. Another reason for discrepancies was the completion of numerous registers by the healthcare workers who already had a high workload and who were required to manually complete the register when there was a long waiting list of clients to attend to [6,7]. This subsequently leads to data entry or transcription errors during data collection and contributes significantly to misrepresentation of the work done by health care workers in the facilities, which eventually impacts future programme planning to improve service delivery [7,8].

Research by Nicol 2015 has demonstrated that adequate training is not usually provided for South African clinicians involved in data-collection processes, who frequently have very limited data quality checking skills and do not understand the value of the data being collected. They are most often not trained on the programmes care pathways and data monitoring, and as such data captured into the Routine Health Information System (RHIS) may be of low quality [8]. Consequently, there are a number of opportunities for transcribing errors, particularly when these tasks are performed in unconducive environments, like the spaces the nurses work in. In addition, the definition of the data elements themselves may contribute to the challenge as in many instances; the denominators and numerators are not matched; that is, the numerators are not a subset of the denominators [8].

The various collection tools at the facility are also a challenge. These collection tools are sometimes not aligned with the DHIS. There are lots of data elements collected at the facility level that are not captured onto the DHIS [3,6]. There are also other elements required in the DHIS that are not on the clinic registers. Often clinics experience stock-outs of registers and the nurses are forced to use sheets photocopied from unused pages of old registers, and may misplace the loose sheets, resulting in data loss. At times the writing on the copied sheet may not be legible, making it difficult for the data capturers to read the information on the sheets during capturing. This will also impact on the quality on data collected. In addition, the definition of the data elements themselves may contribute to the challenge as in many instances; the denominators and numerators are not matched; that is, the numerators are not a subset of the denominators [8].

To have an in-depth understanding of HealthCare Professionals (HCPs) experiences during the transitional phase of implementing the rationalised registers, we conducted an exploratory study on the Prevention of Mother-to-Child Transmission (PMTCT) data management and reporting in Amathole District, Eastern Cape province, South Africa. We also explored the effect of the rollout of ROR on the documentation and reporting of PMTCT programme data with the existing NDoH source documents during the transitional period.

## 2. Methods

A mixed-method research approach was used for this study [1]. First, we reviewed three source documents [Tally sheet, Antenatal care (ANC) register, Tick register] used for collecting and reporting PMTCT data to enable us to document the similarities and differences in the PMTCT data elements reported. After that, in-depth interviews were conducted to understand the HCPs experience on how PMTCT data elements were collected and reported in the facilities during the transitioning phase of the rationalised registers. We also documented the experiences of HCPs on their perspectives on the cause of PMTCT data discrepancies and suggested ways of addressing them. Thereafter, Chi square test was used to analyze two categories of participants (professional nurses and data capturers) opinion about the causes of discrepancies in PMTCT data reporting and what can be done to address the discrepancies at district and sub-district level, with the *p*-value to show the level of significance.

### 2.1. Data Collection

Information on PMTCT data reporting was collected by conducting a mapping exercise of the old and new registers in order to map each data element in the registers with the appropriate PMTCT indicators that are being monitored on a monthly basis. This was done to better understand, identify and correlate the similarities and difference of the registers. Thereafter, the findings were inputted into excel spreadsheet. In addition, we administered questionnaire and conducted in-depth interviews with 24 study participants which were purposively selected [16 professional nurses (4 per sub district) and eight data capturers (two per sub district] across four sub-districts (Amahlathi, Mbhashe, Mnquma and Nkonkobe) of the Amathole district municipality in the Eastern Cape province of South Africa. The facilities supported by the participants include two Community Health centres, 23 Primary Health Care centres, and two Gateway Clinics where they were responsible for documenting and reporting PMTCT data.

Two research assistants were trained on the interview guide and supported via Skype video calls and telephonically to address any issue raised while conducting the study. The research assistants made appointment in good time and at the convenience of the respondent to ensure that the interviews do not disrupt the delivery of essential services. In addition, the research assistants ensured that each participant understood and signed the consent form during the interviews. The participants were informed of the purpose of the study, agreed to participate in the study of their own free will and qualitative interviews were conducted in one of the designated rooms in the facilities.

### 2.2. Data Analysis

The selected eight PMTCT data elements collected during the mapping exercise of the old and new registers with the appropriate PMTCT indicators were entered into a Microsoft Excel spreadsheet and matched against the appropriate source document to show how they are reported, and a summary of the indicators reported using one or more source documents were noted and recorded.

A Chi-square test was conducted to evaluate the difference in the participants opinion (professional nurses and data capturers) on the causes of discrepancies in PMTCT data reporting and the recommended ways of addressing PMTCT data discrepancies. Microsoft Excel was used to conduct Chi-Square test with *p*-value of <0.05 as the level of significance. We collected qualitative data for thematic and textual analysis following the in-depth interviews. Thereafter, we developed codes based on the anticipated themes by reading through the data repeatedly, breaking down the themes, categorising and building them up.

### 2.3. Ethical Consideration

The study was approved and ethically cleared by the Senate Research Ethics Committee of the University of the Western Cape, Cape Town, South Africa. Permission was obtained from the Health Department of the Eastern Cape province. All the participants signed the consent forms voluntarily. The research participants’ identities were protected, and all information was kept confidential.

## 3. Results

The findings of this study were categorized into seven sub sections. The first part (i) is the overview and description of the three source documents used for documenting and reporting PMTCT data across three different levels: health facility, sub district and district. This sub section provides detailed description of each source document and explained what they are used for, (ii) availability of the three source documents in the health facilities and their use for documenting and reporting PMTCT data, (iii) reporting of PMTCT data using the three source documents during the transitioning phase of the rationalized register. The last part (iv) describes how PMTCT data was analysed and used for quality improvement in the facility and presented, (v) participants opinion about the causes of PMTCT data discrepancies (vi) exploring the reasons why some health facilities will not display their facility performance, and (vii) conclude by sharing the recommendations of what need to be done to address the gaps identified and improve the quality of PMTCT data collected.

### 3.1. Overview and Description of the Source Documents for Reporting PMTCT Data

To review the data elements recorded and collected for the PMTCT programme, we analysed the use of three selected DoH source documents available in the facilities for reporting PMTCT data and checked for data reporting alignment.

We found that all the 27 facilities selected for this study agreed to have completed the ANC register, the tick register, and the input forms. After that, they consolidated their PMTCT data monthly before transferring to the DHIS. The nurses completed the tick registers, followed by daily and weekly data verification using the input form and later give it to the data capturers for capturing into the DHIS [9].

- Electronic tick register (e Tick register)

This is a source document used for reporting PMTCT data elements. The standardised paper-based register was transitioned to an electronic tick register (e-tick register) in 2017/2018 during the implementation of the rationalised registers. The register is placed in each consultation room for use by each clinician to complete for each patient daily. The e-ticks run on Libre office, a free and open-source office productivity software suite which enables the collation and consolidation of data by the data capturers before capturing onto the web-based District Health Information Software (webDHIS).

- Antenatal Care (ANC) Register

The source document that is used to record each visit made by a pregnant woman during the antenatal period is the antenatal register. This register is significant because it is the basis on which the quality of antenatal care is retrospectively monitored during delivery. This register documents all events from the registration of the first ANC visit, subsequent visits, pregnancy risk monitoring, routine preventive services offered during pregnancy, and pregnancy outcome details at delivery.

- Input form

The input form is the final form for entering the data into the relevant database. Data from the source documents are collated onto the input forms weekly for reporting into the DHIS using the monthly summary sheet. However, in some cases where there are no input forms, data can still be reported to the DHIS using the monthly summary sheet after data collation. This is done weekly and monthly, after which the data input form is signed off by the facility manager and kept safe under lock in the facility for monitoring and auditing purposes.

Although different data elements are reported using the three source documents listed above, PMTCT data elements may appear in more than one register based on the National Indicator Data Set (NIDS). The NIDS is the minimum number of indicators approved by the NDoH that every public health facility is expected to use to collect, and report based on the facility service package [9]. For this study, we did a mapping exercise of the new registers in order to map each of the eight PMTCT data elements and match them to the appropriate boxes/tables to see how they are reported across the three source documents. Table 1 shows the official source documents for selected eight PMTCT data elements.

PMTCT data elements are part of the core national indicators used to assess the progress and performance of key services provided in PMTCT programme. Data used for reporting are extracted from the DHIS and National Health Laboratory Service (NHLS). PMTCT data elements are important component used for monitoring and measuring the quality of PMTCT programmee and to follow-up and track exposed infants.

From Table 1, we observed that all the three source documents were used to report four of the eight PMTCT indicators (Antenatal 1st visit before 20 weeks, Antenatal HIV 1st test, Antenatal HIV 1st test positive and Antenatal client HIV re-test). In addition, three indicators were reported using two source documents (except the ANC register), while only one indicator (ANC start on ART) was reported using the ANC register alone.

### 3.2. Availability of Source Documents in the Facility

The rationalised registers implementation commenced during 2016/2017 (from April 2016 to March 2017) DOH financial year, but various facilities started the implementation at different times. Therefore, we conducted a desk review and analysis of the 27 facilities to assess the availability of the source documents in January 2017. The availability of the source documents in the facility is important because they are used for recording, collecting, and reporting PMTCT data at the facility level, and this feeds into the sub-district, district, province, and national reports.

**Figure 1:**
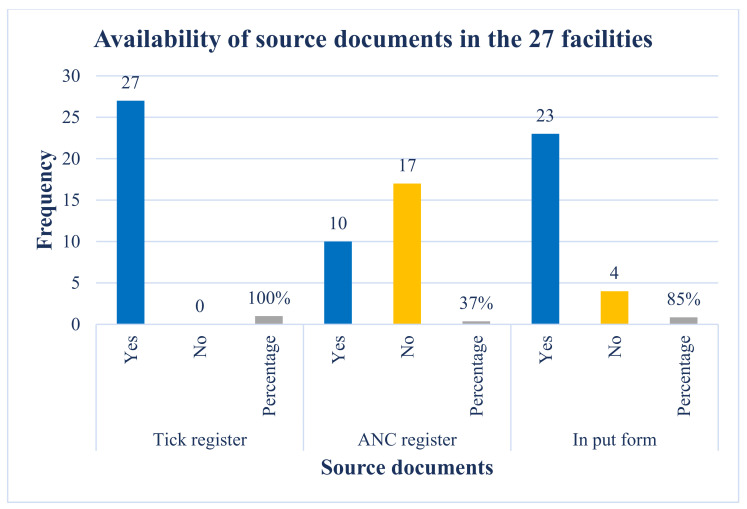
This shows the number and percentage distribution of the three source documents (Tick Register and PMTCT Registers and input forms) across the 27 facilities.

Findings from the desk review of the registers showed that all the facilities (100%) had a tick register for reporting, and only 10 of the 27 (37%) had an ANC register, while 23 of the 27 facilities (85%) had an input form that is used to collate PMTCT data (Figure 1). This shows that the tick register is the main source used as a reliable source document for reporting PMTCT data as at the time of conducting this study. Our study also showed that the tick register is readily available for use in each consulting room in the facility compared to other registers because it is easy to use, does not require the HCPs to do a lot of writing like other registers and therefore they spend less time in completing the register. This correlates well with the findings in Table 1.

### 3.3. Use of Source Document to Report PMTCT Data during the Transitional Phase of the Rationalised Registers

This section reports on the HCPs experience on how PMTCT data was collected, reported, and used in the facility during the transitioning phase of the registers. It further elaborates on the issues encountered in the health facilities that prevent them from not analysing and displaying data in the facility to show their performance.

From the in-depth interview data, less than half of the facilities confirmed that they had already started using the rationalised registers, while the others still use the old registers because they are more comfortable with it. This shows that the DOH need to set up systems that will assist the health facilities to successfully embrace the change management needed to ensure the use of the rationalized registers. Furthermore, the participants attest to not transitioning entirely because they still use some registers, especially the ANC register, stating that

*It is a longitudinal register that captures the pregnant woman’s journey from start to finish*.

(Professional Nurse)

Our data shows that the facility staff work hand in hand to follow the NDoH data collection and reporting process and ensure that data is analysed and captured by the data capturers, verified, and signed off by the Operational Manager before submission.

In the Community Health Centres (CHCs) and PHCs, where the tick register was used for reporting, the nurses complete the tick registers daily and weekly and used the input form for data verification and collation of data for reporting in the DHIS. Once the data has been verified, the data is then given to the data capturers for input into the digital system. Interestingly, all the facilities assessed had at least one data capturer dedicated to capturing PMTCT data in their facility. The excellent coverage of data capturers across the facilities is attributed to support provided to the Department of Health (DoH) by partners which provided additional data capturers to complement the facility staffing structure to improve the data management system.

One facility staff interviewed said,

*Yes, we use the tick register in the counselling and consulting rooms. We use the input forms to consolidate the monthly data for reporting. ……. Here, we (nurses) set aside 30 min at the end of the day to consolidate the number and send it to the data capturer for capturing. All registers are collected, and verification is done daily, before data is captured into DHIS*.

(Professional Nurse)

In agreement with the above, one of the participants stated that

*In my clinic, we started using the rationalised registers in December 2016 because we were told not to use too many registers, but we continue to complete the ANC register, input form and tick register, and they are up to date. The other register needs too much writing, but we prefer the tick register for reporting because it is better*.

(Professional Nurse)

We noticed from the above findings that although a mandate was given to implement and use the rationalised registers, some clinics still hold on to some of the old registers and use them for reporting because they are comfortable using them. However, the tick register remained one of the source documents used for reporting PMTCT data, and the facility staff completed this register according to the Standard Operating Procedure (SOP). This quote by the participants about the preference for using of the tick register over other registers for reporting shows one of the benefits of rationalisation, which is the introduction of a use friendly tick register.

Another respondent stated that

*Each consulting room has a tick register. Every time a patient is seen, it is ticked off in the tick register. The nurses tick the services offered to the patients, and the data capturers put this on the input forms before capturing them on the information system. Everyone is trained on completion of the registers, and we have daily, weekly, and monthly data reviews*.

(Professional Nurse)

From the above participants, we deduced that the facility staff were aware of the requirement to document, capture, and report PMTCT data collected according to the NDoH SOP.

### 3.4. Use of Analysed PMTCT Data Reported

Following the data collection and verification, many facilities drew graphs to analyse the data and display the facility’s PMTCT programme performance in designated areas in their facility. This forms part of CQI in the facility, which is a continuous process of reviewing the progress made with PMTCT programme and assist the facility to refine the programme to perform better. Graphical representation of the indicators was displayed either in the waiting area, on the passage wall or in the operational manager’s office. This gives a quick and effective way of looking up at the charts to know how the facility is performing against the set targets. Across the facilities, three of the six selected PMTCT indicators that were predominantly displayed are first ANC visit before 20 weeks, ANC re-test and ANC clients initiated on ART. In some facilities, other non-PMTCT indicators were displayed alongside the PMTCT indicators.

One of the respondents explained:

*My facility analyses the data and displays the facility’s performance to show what we have achieved with our quality improvement plan. We show data like ANC re-test, ANC clients initiated on ART, ANC first visit before 20 weeks, Isoniazid Preventive Therapy and infants initiated on cotrimoxazole on the walls*.

(Operational Manager)

Another respondent confirmed that the analysed data were displayed on the facility notice board, but the data analysed and displayed are not limited to PMTCT data elements alone.

One of the data capturers confirmed this statement as well,

*The graphs displayed on the notice board or in the offices in my facility are in line with the quality improvement plan we implement. Data analysis is done for all programmes and not specific for PMTCT data alone […] like HIV testing, viral load graph and run charts for ANC visits before 20 weeks*.

(Data Capturer)

### 3.5. Causes of PMTCT Data Discrepancies

More than half of the facilities assessed affirmed that data collection and verification is done in their facilities and the facilities display the PMTCT programme performance and review the progress made as part of continuous quality improvement. However, PMTCT data discrepancies still occur in facilities where the factors detailed in Table 2 below have not been addressed as seen in the analysis of the questionnaire administered to the 16 professional nurses and 8 data capturers on their opinion on the causes of PMTCT data discrepancies.

From our data analysis, data entry error was highly significant, while inadequate training of M and E staff and nurses not documenting their work done properly were significant at *p*-value of <0.05, while the participants opinion about missing source documents (ANC, tick register & Input forms) was not significant.

### 3.6. Reasons for Not Displaying Facility Performance

Although many of the facilities displaced their facility performance in conspicuous area in the facility, others did not. The two main reasons reported for not displaying facility performance include clinicians not properly completing the clients’ information, especially on the tick register and a shortage of staff to collect, report, and analyse data. Facility staff interviewed raised the issue of clinicians not recording or documenting the service rendered properly in the register. Others mentioned instances where the clinicians had recorded incomplete information of the clients they had seen, and this was attributed to the high volume of patients seen daily in the facilities with the low number of HCPs that are available to consult them. In addition, few data capturers affirmed that they are sure that clinicians provide healthcare services to more clients than those indicated or recorded in the register and that facility performance would improve if the clinicians could tick off the work done on the input forms for every client seen. This finding is in line with the Chi-square analysis of the participants opinion about the causes of discrepancies in PMTCT data reporting, which showed a significant relationship in two categories relating to the causes of discrepancies in PMTCT data. They are nurses not documenting work done properly at *p*-value of 0.003770 (*p*-value < 0.05) and data entry error at *p*-value of 0.000347 (*p*-value < 0.001) as seen in Table 2.

Another challenge at the facility level is that doctors and nurses may forget to capture information on every patient that presents for care as a result of the multiple roles they play (e.g., provision of clinical care and completion of registers) and high volumes of patients. These may lead to under or over reporting. It can therefore be difficult to keep accurate statistics, and this might not give a true reflection of care given in the facility.

The following are quotes from the participants highlighting some of the reasons for poor documentation of records

*We do [not] display any PMTCT graph although we analyse the PMTCT dashboard monthly […] we do data analysis for all programmes and capture our data on the District Implementation Plan (DIP) template. Still, some nurses do not complete their registers very well*.

(Professional Nurse)

*Discrepancies and inconsistencies in PMTCT data collated and reported in my clinic are due to nurses forgetting to tick the register, sometimes resulting in data not corresponding with the work done. We see minor discrepancies in some of the columns in the tick register because the nurses did not tick them or because they did not record properly in the register even when the service was offered. As data capturers, we assumed that the service was provided although it was [not] indicated in the register. This results in challenges in data collection during data capturing at times*.

(Data Capturer)

From the above statements, we note that the importance of good documentation (recording and reporting of work done) cannot be overemphasised for reporting and representing the work done by the facilities.

In addition, the other point made by the staff interviewed was the staff shortage issue. The perception was that, although they collect, review, and analyse their data, they did not have the capacity to generate nor display the graphs that show their facility performance because they do not have experts assigned to support the facility.

One of the participants states:

*We do not display graphs in my clinic because of a shortage of staff and also because we don’t have anybody from DOH to do the graph for us. If our partner is not around, we can’t do it. My facility has challenges in data collection and data capturing. More hands are needed because the clinic is short-staffed and not properly supported. We also need more support and supervision from the sub-district*.

(Operational Manager)

### 3.7. Suggested Ways to Address Gaps Identified in PMTCT Data Collected

Following identifying gaps in the facilities, we sought the participants’ opinions on a possible solution to address the gaps identified. The following suggestions were made (i) dedicating more time to identify and understand the reasons for the gaps identified in the data collected, (ii) addressing shortage of staff issue, (iii) providing supportive supervision and (iv) capacity building of staff, especially the data staff. This corresponds to the Chi-square analysis, which suggested that it is important to build the capacity of M and E staff and have designated staff responsible for reviewing data quality at facility and sub-district levels with *p*-values of 0.000347 and 0.045500, respectively (*p*-value < 0.05) in Table 3 below.

Our data analysis about the participants opinion in Table 3 showed that capacity building of M and E staff is highlight significant, having designated staff responsible to review the quality of PMTCT data at facility and sub-district level was significant while the other two factors; having written guidelines from M and E unit for facility reporting and ensuring that all M and E positions are filled and dedicated to data management are not significant.

*We can correct discrepancies and inconsistencies in data collated and reported by ensuring that all gaps are identified during data verification to help us address all discrepancies during our weekly meeting. We need to have weekly meetings in the facility to check data discrepancies [constantly] and identify gaps to improve the quality of data such as providing more staff to assist us.*

(Professional Nurse)

Another participant stated that:

*I think we can address these challenges by investing more time in improving our data and building the capacity of data capturers, particularly on topics such as PMTCT. We need to emphasise adherence to the facility policies. We also need to think about the consistent use of the registers, get rid of notebooks and consolidate our [statistics] daily. Other things that we can do is data verification every month, and to strengthen the support provided to the facility [and] the data management at the facility level. We need more of capacity building and clinic supervision*.

(Operational Manager)

Our study showed that the facilities selected complete the three source documents used for recording and reporting of PMTCT data elements with preference for the tick register because it is user friendly, which is line with the purpose of scaling up the rationalized registers. However, some of the facilities have not transitioned completely to using the rationalized registers because they are so used to the old registers. In addition, the participants agreed that it is important to conduct data verification, display the PMTCT programme performance and review programme data as part of the continuous quality improvement process in the facilities. Therefore, there is need to address the gaps identified in PMTCT data collection process.

## 4. Discussion

From our findings, recording of data elements in the registers and ensuring that the PMTCT data reported to the District Health Information System is of good quality is key for successful implementation of the ROR. In addition, it is important to understand the District Health Management Information System policy which can guide and minimize data discrepancies. There is also the need to implement change management to ensure that the remaining facilities that have not transitioned to using the rationalized registers do so.

The District Health Management Information System (DHMIS) Policy of 2011 [9] was designed to provide comprehensive, timely, reliable, and good-quality routine data. To accomplish this, nurses must complete daily tally sheets at the facility for all clients and submit weekly tally sheets (input forms) to the data capturers for them to manually collate the data elements into weekly summary sheets. This data is then compiled into a monthly summary sheet and signed off by the facility manager [6].

### 4.1. Causes of Data Discrepancies and How Facilities Are Addressing Them

From the participants’ responses, poor documentation, inadequate training of M and E staff and data entry errors were the three main reasons for data discrepancies. This shows that there is a need to address these challenges in order to achieve the purpose of the ROR, which were to improve data quality and inconsistencies in reporting by empowering the health professionals and ensuring that they complete the registers correctly, collect data and report them appropriately. From our findings, capacity building of health professionals, institutionalizing change management and monitoring the implementation of the rationalised registers are essential factors required for successful scale up plan as seen in studies from other countries. This was demonstrated in Indian by Antara Foundation, where a similar project in partnership with the Ministry of Health in Rajasthan, Indian, was implemented in 2017 through the Akshada programme [10]. This project focused on the rationalisation of the Auxiliary Nurse Midwife (ANM) service delivery register, which was designed to be user-friendly with a clear role for the users, and at the same time focused on meeting the government reporting needs. The overall goal was to reduce the workload of the ANM without losing essential information and create more time for healthcare workers to provide quality service delivery [11].

Young et al. [12] conducted a retrospective analysis of matched test results from Mozambique’s PMTCT HIV testing programme. One of the reasons for discrepancies in data was incorrect entry or transcription of data during data collection or when ANC visits were captured centrally. The study thus recommended that double entry of data and automatic transcription of results be implemented to address this issue [13]. In like manner, a study conducted in Kenya on PMTCT data accuracy found that nurses admitted that they sometimes forget to record the services provided to patients [3], this finding is similar to what was found in this study where one of the causes for PMTCT data discrepancies was nurses not documenting the work done properly which was significant at *p*-value of 0.003770 (*p* < 0.05). Specifically, nurses reported that despite recording the woman’s HIV status in a client-held booklet provided during the first visit to ANC, they did not record whether prophylaxis was provided or not.

### 4.2. Addressing Gaps Identified in PMTCT Data Collection

According to the participants interviewed, more time needs to be dedicated to identifying the gaps in data collection and providing staff to fill vacant positions, especially in facilities located in the rural areas where it is difficult to retain skilled staff and ensure that the full staff complement according to the establishment list is in place. These staff also require training and supportive supervision to enable them to close the gaps in accurately documenting PMTCT services. The participants specifically highlighted the need for the facilities to be adequately staffed with data capturers and to support PMTCT data collection. In their article, Mutshatshi et al., agree with this statement, stating that it is problematic if records are not kept satisfactorily within the facilities [11]. In addition, the authors recommended that there is a need for continuous training of clinicians and follow-up to address challenges encountered in order to improve patient management and care.

### 4.3. Possible Solutions to Address Data Discrepancies

The main point for addressing data discrepancies is to hold everybody in the facility responsible for documenting and accounting for all the work they have done and to review the facility-level data and ensure that quality data is submitted to the sub-district and district levels.

This finding is in line with the recommendations in the data management section of the National PMTCT 2019 guidelines published by the South African by NDOH [14], where clinicians are required to document all clinical findings, results, and decisions clearly in the source documents such as the maternity case record, adult clinical record (ART stationery) for HIV positive women, and in the Road to Health Booklet (RTHB) for the HIV-exposed infant [8]. Likewise, the facility level standard operating procedure for district health management information system [15] states that health care providers (nurses, doctors, and other health professionals) are responsible and accountable for ensuring that there is high-quality data in individual patient files and that routine data are collected and collated using the standardised registers and other appropriate data collection tools. In addition, all health care providers must record individual patients’ data in the patient retained records during or directly after each patient consultation [15].

In addition, the importance of having a good system in place to collect and report good quality data cannot be over emphasized as part of the continuous quality improvement process at all levels of health care delivery. Therefore, there is need to address the gaps identified in PMTCT data collection process. There is need to scale up quality improvement initiative led by DOH in collaboration with partners which was aimed at developing, implementing and monitoring a data-driven intervention to improve the quality of facility, district, provincial and national PMTCT data collection and reporting process. For this intervention to work better, efforts should be made to ensure that a series of critical pathways called the PMTCT cascade is followed, where the journey of a pregnant HIV-positive women is monitored as she moves through the different stages on antenatal care in order to receive appropriate care and treatment for themselves and their new-borns [16,17]. This is monitored through the use of process indicators collected routinely as part of the DHIS as done in some other countries [18,19,20,21]. Additionally, data quality needs to be addressed through mechanisms incorporated into the data collection process and functions within the DHIS software. These include checking of the data for inaccuracies by clinic managers and supervisors, using minimum and maximum expected values for each data element collected, and using the DHIS software. The minimum and maximum values for a data element are calculated on the basis of previous experience at the facility, outliers are identified and possible reasons for these are accounted for. Rigorous data deep dive is explored to identify inconsistencies and data errors, including programmatic underperformance to institute corrective actions [22].

Lastly, WHO [23] highlighted the importance of validating and improving the national PMTCT programme data. One of the recommendations made was to measure the impact of PMTCT programmes using retrospective or prospective cohort data linked to PMTCT intervention data and programme using ANC or birth cohort [24]. Two examples that described the use of birth cohort reporting were documented in Kenya and Malawi where the facility used the HIV exposed infant longitudinal follow-up register to monitor infants by birth-month cohorts up to 24 months as well as the use of reporting forms to follow up HIV-exposed infant, respectively [23].

Our study has shown that the implementation of the rationalized registers has raised the awareness about the importance of institutionalizing a good system that will ensure that the facilities are reporting good quality data and using the data to improve performance as part of continuous quality improvement. In order for this to be done successfully, there is need to address the causes of PMTCT data discrepancies such as capacity of the clinic staff involved in data collection and implementation of change management process to ensure that the changes made about the transitioning of the registers from 56 to 6 is implemented and the health care professionals accept the use of six registers. This can be done by adequately preparing and supporting them with the necessary steps they need to undergo for the change to be successful and ensuring that there is monitoring process post implementation.

### 4.4. Study Limitations

Our study was conducted in Amathole district of Eastern cape province, South Africa, which is one of the 52 district municipalities in South Africa; therefore, our participants perspectives are only limited to the district. We recommend a need for a study that will include all the 52 districts in South Africa in the future to give a better understanding and perspectives of other study participants across the country which will explore the acceptability, efficiency and effectiveness of the rationalized registers across the country.

## 5. Conclusions

PMTCT data management and reporting were challenging during the transitioning phase of implementing the rationalised registers because of different timelines instituted in the facilities and the non-availability of source documents in some facilities. The big challenge of routinely collected PMTCT data occurs at the facility level, affecting data reported at the sub-district/district and national levels.

The primary source of inaccuracy lies in the data collection at health facilities, with wide discrepancies between data in the source documents and on the summary sheet rather than the transcription of the data from the summary sheet into the software system [6,9]. This can be attributed to clinicians not reporting all the work they do, and inadequate monitoring of the PMTCT indicators through data verification and validation, which affects the data reported and overall performance of the facility, sub-district, district and national levels.

We recommend that the capacity of the clinic staff involved in data collection should be built not only on programme care pathways but on data monitoring and data capturing into the Routine Health Information System (RHIS), consistent review of facility-level data to ensure that quality data before reporting, and deep-dive analysis and data use for programme improvement. This should be complemented with coaching, mentoring, and supportive supervision, in addition to closely monitor the progress made for improved programme outputs and outcomes. However, it has been shown that the ROR has potential for to improve data quality.

## Figures and Tables

**Table 1:** Official source documents for selected eight PMTCT data elements.

	Data Element	Indicator Definition	Tick Register	ANC Register	Input Form
I	Antenatal 1st visit before 20 weeks	A first visit by a pregnant woman to a health facility that occurs before 20 weeks after conception to primarily receive antenatal care according to Basic Antenatal Care (BANC). The first antenatal visit is often referred to as a ‘booking visit’	X	X	X
II	Antenatal HIV 1st test	Antenatal client who was tested for the first time during her current pregnancy	X	X	X
III	Antenatal HIV 1st test positive	Antenatal clients who tested positive for the first HIV test done during the current pregnancy	X	X	X
IV	Antenatal client HIV re-test	Antenatal clients who tested negative for HIV during an earlier antenatal visit and were re-tested for HIV during the pregnancy	X	X	X
V	Antenatal start on ART	HIV positive antenatal clients who were initiated on ART during their current pregnancy		X	
VI	Infant PCR test around 10 weeks	Infants born to HIV positive mothers who are PCR tested around 10 weeks	X		X
VII	Infant PCR test positive around 10 weeks rate	Infants tested PCR positive for follow up test as a proportion of Infants PCR tested around 10 weeks	X		X
VIII	Infant initiated on CPT around 6 weeks	Children under 15 years who were on cotrimoxazole prevention therapy (CPT) at the time of starting ART	X		X

X indicate that the source document to report the PMTCT data element.

**Table 2:** Major causes of PTMCT data discrepancies.

Inadequate Training of Monitoring and Evaluation (M and E) Staff						
	Group 1 (16 PNs)	Group 2 (8 DCs)	Total	Chi-Square	*p*-Value	Remarks
Yes	13	1	14	10.37142857	0.00128	*p*-value < 0.05 Significant
No	3	7	10			
Total	16	8	24			
Nurses not Documenting Work Done Properly						
	Group 1 (16 PNs)	Group 2 (8 DCs)	Total	Chi-Square	*p*-Value	Remarks
Yes	4	7	11	8.391608392	0.003770	*p*-value < 0.05 Significant
No	12	1	13			
Total	16	8	24			
Missing source documents (ANC, Tick Register & Input Forms)						
	Group 1 (16 PNs)	Group 2 (8 DCs)	Total	Chi-Square	*p*-Value	Remarks
Yes	12	8	20	2.4	0.121335	*p*-value > 0.05 Not significant
No	4	0	4			
Total	16	8	24			
Data entry Error						
	Group 1 (16 PNs)	Group 2 (8 DCs)	Total	Chi-Square	*p*-Value	Remarks
Yes	14	1	15	12.8	0.000347	*p*-value < 0.001 Highly significant
No	2	7	9			
Total	16	8	24			

**Table 3:** Participants opinion about what can be done to address PMTCT data discrepancies.

Capacity Building of M and E Staff						
	Group 1 (16 PNs)	Group 2 (8 DCs)	Total	Chi-Square	*p*-Value	Remarks
Yes	14	1	15	12.8	0.000347	*p*-value < 0.001 Highly significant
No	2	7	9			
Total	16	8	24			
Written Guidelines from M&E Unit for Facility and Sub District for Reporting						
	Group 1 (16 PNs)	Group 2 (8 DCs)	Total	Chi-Square	*p*-Value	Remarks
Yes	14	6	20	0.6	0.438578	*p*-value > 0.05 Not significant
No	2	2	4			
Total	16	8	24			
All M and E positions dedicated to data management need to be filled						
	Group 1 (16 PNs)	Group 2 (8 DCs)	Total	Chi-Square	*p*-Value	Remarks
Yes	15	5	20	3.75	0.52808	*p*-value > 0.05 Not significant
No	1	3	4			
Total	16	8	24			
Have Designated Staff Responsible for Reviewing the Quality of PMTCT Data at Facility and Sub-District Level						
	Group 1 (16 PNs)	Group 2 (8 DCs)	Total	Chi-Square	*p*-Value	Remarks
Yes	14	4	18	4	0.045500	*p*-value < 0.05 Significant
No	2	4	6			
Total	16	8	24			
